# Short-term Efficacy of Micropulse Yellow Laser in Non-center-involving Diabetic Macular Edema: Preliminary Results

**DOI:** 10.4274/tjo.04657

**Published:** 2018-10-31

**Authors:** Mehmet Fatih Kağan Değirmenci, Sibel Demirel, Figen Batıoğlu, Emin Özmert

**Affiliations:** 1 Ankara University Faculty of Medicine, Department of Ophthalmology, Ankara, Turkey

**Keywords:** Micropulse, laser, diabetic macular edema

## Abstract

**Objectives:**

The aim of this study was to evaluate the efficacy of micropulse yellow laser (MPL) on best corrected visual acuity (BCVA) and retinal thickness in patients with non-center-involving diabetic macular edema (DME).

**Materials and Methods:**

We retrospectively reviewed 9 eyes of 8 patients with non-center-involving DME who underwent MPL treatment between January 2015 and December 2016. BCVA (logMAR) and retinal thickness were evaluated before and 3 months after treatment. Maximum retinal thickness was determined manually from simultaneous spectral-domain optical coherence tomography images and recorded. The change in the measurements from before to after treatment was analyzed statistically.

**Results:**

Of the 8 patients, 3 were female and 5 were male. The mean age was 52.8 years. Two of the 9 eyes had received previous intravitreal anti-vascular endothelial growth factor injection(s). Median BCVA was improved 3 months after treatment, although the difference was not statistically significant (0.34 logMAR before and 0.29 logMAR after treatment). BCVA was improved in 4 eyes while it showed no change in the remaining 5 eyes. The mean retinal thickness was 470.6 μm at baseline and 416 μm at 3 months after MPL treatment (p=0.01). Retinal thickness decreased in all eyes after treatment.

**Conclusion:**

In this study, parafoveal retinal thickness showed significant decrease after MPL treatment in patients with DME. The limited increase in BCVA may be due to the inclusion of a low number of patients and only those with non-center-involving macular edema. MPL may be used as an alternative to conventional argon laser in non-center-involving DME.

## Introduction

Diabetic macular edema (DME) is the most common cause of vision loss in patients with diabetic retinopathy (DRP). Thermal laser photocoagulation has long been used as the standard treatment for clinically significant DME.^1^ Despite its therapeutic effectiveness, it can lead to undesirable complications such as visual field loss, choroidal neovascularization, epiretinal fibrosis, and enlargement of laser scars.^2,3,4^ Micropulse laser (MPL) is a method developed to reduce the laser-induced thermal damage caused by conventional laser therapy.^5^ In the micropulse mode, laser is applied in short pulses, thereby reducing the thermal energy generated in the target area.^6^ The coagulation scars seen after conventional laser application do not form with MPL treatment.^7^ Today, intravitreal anti-vascular endothelial growth factor (anti-VEGF) injection has been embraced in the treatment of DME, and its efficacy has been reported in several studies.^8,9,10^ However, in some cases the expected functional/anatomical success is not achieved with anti-VEGF administration.

The aim of this study was to investigate the effect of yellow (577 nm) MPL therapy on best corrected visual acuity (BCVA) and retinal thickness in patients with parafoveal macular edema that does not involve but threatens the central macula.

## Materials and Methods

Ethics committee approval for the study was obtained from the Ankara University Faculty of Medicine Clinical Research Ethics Committee (20-1249-17). The study was carried out in accordance with the tenets of the Declaration of Helsinki. The study included 9 eyes of 8 patients who were being followed for DRP in our retina outpatient clinic, had macular edema that did not involve but threatened the fovea, and underwent MPL therapy with a 577 nm yellow laser (Supra Scan, Quantel Medical, Cedex, France). A single-spot test shot in micropulse mode was applied to a non-edematous area of the macula outside the temporal vascular arcade. The laser power was gradually increased until it formed a faint laser spot. The power of the micropulse pattern laser was set at 50% of the power needed to form a barely visible laser spot. Laser parameters used were 200 ms duration, 160 µm spot diameter, low operating cycle (5%), and high density (contiguous laser spots). Optical coherence tomography (OCT) thickness maps were consulted to select the most suitable scanning pattern for the entire edematous area. BCVA (logMAR) was recorded before and 3 months after the treatment. Spectral-domain OCT (SD-OCT) and fundus autofluorescence (FAF) images were obtained at the same time points. The point of greatest retinal thickness was determined manually, and measurements were recorded. Pre- and post-treatment FAF images were compared in terms of laser-induced scar formation. The differences between pre-treatment and month 3 post-treatment median BCVA and mean retinal thickness were statistically compared using paired samples t-test.

## Results

Of the 8 patients included in the study, 5 were male and 3 were female. Their mean age was 52.3 years. All 9 eyes were evaluated as having non-center-involving parafoveal macular edema using SD-OCT images. Two of the eyes had previously received intravitreal anti-VEGF therapy. Of these 2 eyes, 1 had received 4 anti-VEGF injections and the other had received 5 anti-VEGF injections. Both eyes underwent MPL treatment at least 3 months after the last injection. The other 7 eyes with DME had not received any previous treatment, and MPL was applied as initial therapy.

**Figure 1:**
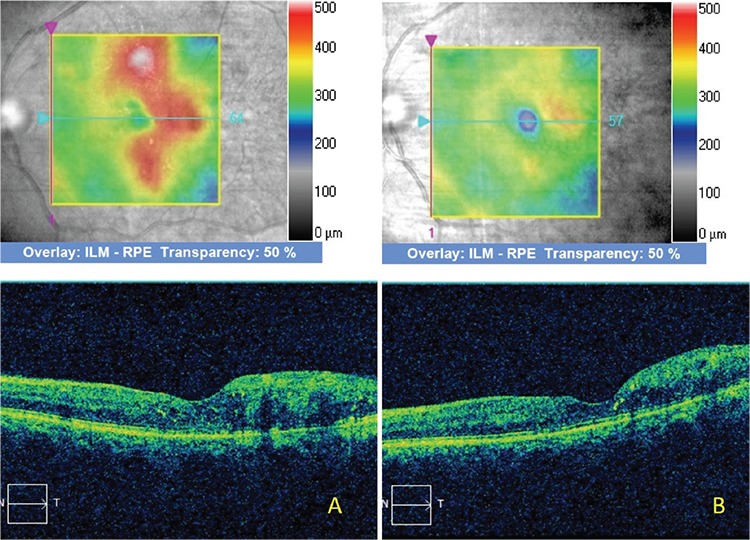
A) Optical coherence tomography images prior to micropulse laser treatment show retinal thickening in the temporal parafoveal area; B) optical coherence tomography images obtained 3 months after micropulse laser

**Figure 2:**
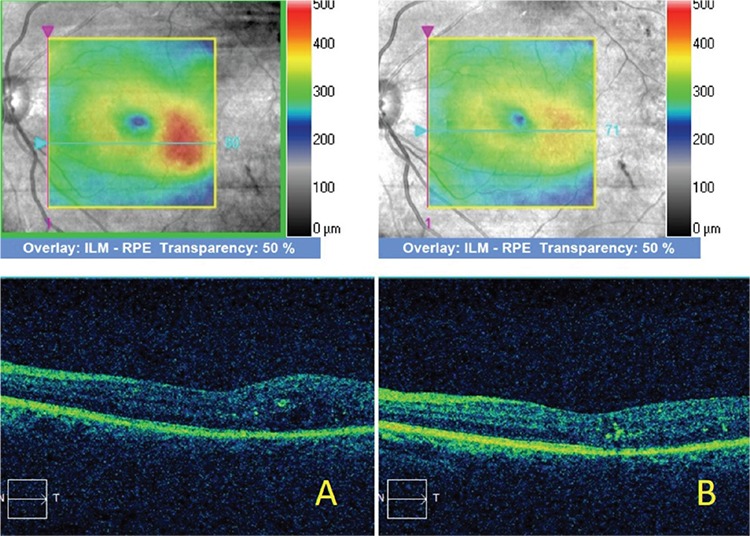
A) Optical coherence tomography images prior to micropulse laser treatment show retinal thickening in the temporal parafoveal area; B) optical coherence tomography images obtained 3 months after micropulse laser

Median BCVA was 0.34 logMAR before treatment and 0.29 logMAR at 3 months after treatment. BCVA increased after treatment in 4 eyes and remained unchanged in the other 5 eyes. However, the increase in BCVA was not statistically significant (p=0.16). In the measurements made manually from the point of greatest parafoveal retinal thickness on SD-OCT images, mean retinal thickness was 470.5 µm before treatment and 416 µm at 3 months after treatment. Retinal thickness had decreased in all 9 eyes at 3 months after treatment (Figures 1a, b and 2a, b). The decrease in mean retinal thickness was statistically significant (p=0.01).

## Discussion

The prevalence of diabetes mellitus is increasing rapidly worldwide. The main cause of vision loss in this patient group is DME. 

Numerous systemic and local factors have been identified in the development of DRP and DME. One of these is the role of the retinal pigment epithelium (RPE). Electron microscope images obtained in diabetic human and animal studies have demonstrated cellular and subcellular damage in the RPE.^11,12^ In addition, diabetes-related changes in RPE permeability and subsequent increase in fluid leakage has been reported in diabetic human and animal models.^12,13^ The RPE releases a number of growth factors, anti/pro-angiogenic factors, and neurotrophic factors, some of which are known and others of which have been newly demonstrated in recent studies. Upregulation of VEGF occurs under hypoxic conditions.^14,15^ VEGF levels in the aqueous and vitreous fluids are known to be correlated with DRP severity, retinal neovascularization, and edema formation.^16^ In addition to the RPE, VEGF is also produced by Müller and ganglion cells. In fact, VEGF production from the neurosensory retinal tissue was shown to have a greater role in DRP than the RPE.^17^

Conventional laser photocoagulation has long been used in the treatment of DME, despite lacking a full understanding of its mechanism.^1^ Unfortunately, this treatment has adverse effects in both the short and long term. Today, the standard treatment method for DME is intravitreal anti-VEGF injection, which is proven safe and effective.^8,9,10^ Laser photocoagulation is still used for edema that does not involve the fovea or is resistant to anti-VEGF therapy.

Besides the pro-angiogenic VEGF molecule, another target in the treatment of macular edema is the RPE cells, which form the outer blood-retina barrier and incur damage and impairment of normal functions in diabetic patients. In the conventional laser procedure, laser light is absorbed by the RPE, resulting in cell damage. This is believed to reduce VEGF production in the RPE as well as decrease retinal oxygen demand and retinal hypoxia.^18^ New laser methods are being investigated in order to reduce the side effects of laser application and increase the effectiveness of treatment.

It was observed in our study that following yellow wavelength (577 nm) MPL therapy in patients with non-center-involving DME that did not require anti-VEGF therapy, BCVA was preserved and/or increased and retinal thickness decreased significantly in the short term. Kwon et al.^19^ applied yellow MPL therapy to 14 eyes with DME with foveal involvement and reported significant improvements in BCVA and central macular thickness at the end of a mean 7.9-month follow-up period. In another study, yellow MPL was applied to 26 patients and infrared MPL was applied to another group of 27 patients with center-involving DME. The eyes were evaluated before and after treatment using SD-OCT, FAF, fluorescein angiography, and microperimetry. No difference was reported between the groups in terms of morphological and functional safety and efficacy after treatment.^20^ In our study, no change was observed in FAF images taken before and at 3 months after the laser procedure. In a study by Inagaki et al.^21^ including 53 eyes with DME, some were treated with yellow MPL while others were treated with 810 nm MPL treatment, and the authors reported that macular edema was decreased, visual acuity was preserved, and the need for additional treatments during the 12-month follow-up period had decreased in both groups.

MPL seems to be very advantageous compared to conventional laser treatment, especially in terms of side effects. In this relatively new technique, the photothermal effect is applied to the RPE in a more controlled way compared to conventional laser. As laser light is continuously applied in conventional laser treatment, tissue temperature increases rapidly, causing permanent photothermal damage to the neurosensory retina. In the MPL method, however, energy is delivered in repetitive “on”-“off” cycles. The short duration of laser light emission limits the increase in temperature, while the longer “off” period enables the reduction of tissue temperature, thus preventing thermal damage.^22^ There is still no consensus on the ideal operating parameters for MPL. However, there are 2 methods of calculating laser power that are generally adopted in clinical practice. In the first method, the power of micropulse pattern is determined as 50% of the laser power that forms a barely visible spot in a single shot in micropulse mode. In the second method, laser power is determined as twice the power that forms a faint burn in a single shot in continuous mode. In the literature, laser parameters used in previous studies include operating cycle of 5%-15%, application time of 100-300 ms, and spot diameter of 100-200 µm, and no evidence of the superiority of any of these settings over the others has been reported.^23^

### Study Limitations

The small number of patients, short follow-up period, and lack of a control group comprise limitations of our study. The limited increase in BCVA may be due to the small number of patients and the inclusion of eyes without foveal edema.

Prospective studies comparing MPL to anti-VEGF therapy in large patient groups and with long follow-up periods are needed to demonstrate the effectiveness and reliability of MPL in DME.

## Conclusion

According to the results of our study, MPL can be considered as an alternative to conventional argon laser for the treatment of non-center-involving DME that threatens the central macula.
